# Improved Gravitation Field Algorithm and Its Application in Hierarchical Clustering

**DOI:** 10.1371/journal.pone.0049039

**Published:** 2012-11-16

**Authors:** Ming Zheng, Ying Sun, Gui-xia Liu, You Zhou, Chun-guang Zhou

**Affiliations:** College of Computer Science and Technology, Jilin University, Changchun, P.R. China; Queen's University Belfast, United Kingdom

## Abstract

**Background:**

Gravitation field algorithm (GFA) is a new optimization algorithm which is based on an imitation of natural phenomena. GFA can do well both for searching global minimum and multi-minima in computational biology. But GFA needs to be improved for increasing efficiency, and modified for applying to some discrete data problems in system biology.

**Method:**

An improved GFA called IGFA was proposed in this paper. Two parts were improved in IGFA. The first one is the rule of random division, which is a reasonable strategy and makes running time shorter. The other one is rotation factor, which can improve the accuracy of IGFA. And to apply IGFA to the hierarchical clustering, the initial part and the movement operator were modified.

**Results:**

Two kinds of experiments were used to test IGFA. And IGFA was applied to hierarchical clustering. The global minimum experiment was used with IGFA, GFA, GA (genetic algorithm) and SA (simulated annealing). Multi-minima experiment was used with IGFA and GFA. The two experiments results were compared with each other and proved the efficiency of IGFA. IGFA is better than GFA both in accuracy and running time. For the hierarchical clustering, IGFA is used to optimize the smallest distance of genes pairs, and the results were compared with GA and SA, singular-linkage clustering, UPGMA. The efficiency of IGFA is proved.

## Introduction

Large-scale gene sequencing technologies [1] such as mRNA micro-arrays have sharply increased our ability to explore the genes organism. To identify genes of interest, we need some algorithms capable of selecting and screening candidate genes for further investigation. Hierarchical method [2] is one method, which returns a hierarchy of nested clusters, where each cluster (subtree) typically consists of the union of two or more smaller clusters. The hierarchical clustering is the notion that similar genes are assigned to the same set using a measure of Similarity.

Two challenging tasks of various optimization algorithms are to search the global optimum and to find all local optima in the solutions space of genes hierarchical clustering from available experimental data, especially from large-scale gene expression data. Selection or creation a proper optimization algorithm is one important work for many system biologists. And a lot of optimization algorithms had been proposed and applied in many biology fields. In these researches, the research on heuristic search algorithms is the fastest growing field. These algorithms include genetic algorithm (GA) [3], simulated annealing(SA) [4], particle swarm optimization(PSO) [5], and even the new algorithm gravitation field algorithm(GFA) [6]. These algorithms were used to optimize a certain function from various obtained data or given system biology problems, such as gene clustering [7], gene regulatory network reconstruction [8], etc.

In these algorithms, the efficiency of GFA, which is a novel heuristic search algorithm proposed in 2010, had been proved for many functions and problems. And some advantages can be found in GFA. First of all, GFA can not only deal with global extreme optimal problems, but also the multi extremes optimal problems which traditional heuristic search algorithms can’t deal with. Secondly, GFA can be convergent in the global solution space with probability 1 in three conditions for object functions of one-dimensional independent variable. And the convergence had been proved through mathematical demonstration [6].

But actually, GFA are not matured as a novel algorithm, especially in two parts. The first part is the theory of GFA. Some immature theory problems should be resolved, especially the strategy of solution space division. The other part is the accuracy of GFA. Some algorithm steps should be improved, and the rotation factor is proposed in this paper to increase the efficiency of GFA. In our prophase research, no effective method can be used for division, only one or two-dimensional variables could be divided properly in solution space. When the dimensionality is greater than two, only one selected dimensionality is used as criteria for division. And other dimensionalities are not considered as the criteria. Some better division strategy should be proposed to improve the division theory quality. In addition, the movement operator of GFA is not good enough for most problems, although the convergence is proved. When the number of dusts is not big enough, GFA will not be convergent with probability 1. New mechanism should be used in this step of GFA.

In this paper, an improved GFA was proposed with two improvements, which is called IGFA. The two improvements are random division and rotation factor respectively. Random division strategy is used for multi-dimensional problem. This method will allocate every dust randomly to any group for certain solution space. And any group will not be dismissed until the optimum in IGFA is not changed for a long time. When all dusts assemble together in one group, the assembled aggregate will be considered as a new dust for next random division. The other improvement is the rotation factor which is used to improve the efficiency of IGFA in this paper and to avoid the local convergence in IGFA. When the small surrounding dusts come to the bigger centre dust, the surrounding dusts could be pushed away from the centre dust in some direction with a certain probability. The whole modified procedure of IGFA will be described in this paper.

In some computational biology, such as reconstruction of gene regulatory network and simulation of gene expression [9], the data used to be analyze are continuous. But in other fields of computational biology, such as hierarchical clustering, the data may be discrete. IGFA can be used to analyze continuous data. So IGFA will be modified again to resolve discrete data problem. Two parts were modified in IGFA. The first one was the dusts initialization in discrete solution space. The other one is the movement operator which concludes four steps to move the dusts and search optimal solution.

Two kinds of experiments from a suite of benchmark functions were used to test the efficiency of IGFA. One is the global minimum searching. The other is the multi-minima searching. And we also compared the performance of IGFA with GFA, GA and SA. 500 minimization runs were used in this paper. The results showed that the performance of IGFA is better than GFA in many cases, including the accuracy and running time.

And for the application in hierarchical clustering algorithm, Yeast Saccharomyces cerevisiae gene expression data were used in this paper. And the results were compared with GA and SA methods. The efficiency of IGFA can be proved.

## Methods

### Brief Introduction of GFA

GFA is derived from the point of the hypothesis theory Solar Nebular Disk Model (SNDM) [10]. The algorithm goal is to search the optimal solution of given function or problem. To start with, all the solutions, which are the dusts in the algorithm model, are initialized randomly, or based on the prior knowledge. What’s more, we assign every dust (solution) a weight, we call it mass, whose values are based on the mass function generated from the criteria function. Finally, the power of the dust attraction, which belongs to a certain dust and exists between every two dusts, pulls other dusts to the dust. Hence, the dusts assemble together, and the planets come out in the end they are the optima. The mathematical proof demonstrates that GFA could be convergent in the global optimum by probability 1 in three conditions for one independent variable mass functions [6].

### Description of IGFA

#### Initialization

IGFA will start with dusts initialization which simulates the dark nebular in SNDM. For continuous data, the task is to generate N dusts 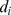
(i = 1,2, …, N) in certain solution space. Any dust 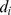 is an M-dimensional vector variable. Many distribution functions could be used as the dusts distribution criteria in this step. Uniform distribution [11] is the most commonly used one. The initial procedure is described below.

Any-dimensional value of dust 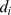
(i = 1, 2, …, N) is generated randomly from the scale region [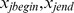] which is the value domain of dimension j in dust 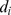. After M times generating operations, 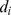 will be one solution in the solution space. N is an important adjustable parameter in IGFA. When N is infinite, the algorithm could be convergent in the global solution space with probability 1 in three conditions for one independent variable mass functions [6]. When N is 1, IGFA will not run at all. So the selection of N should be related to the balance between accuracy and speed with certain problem.

It will be meaningless for the value of 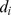 if there is no object function defined in IGFA. 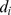 is just defined as a certain location value in the solution space. The object function should be used to decide which dust value is better than others in some standards. The object function in IGFA is called mass function.

#### Strategy of division

The random division strategy is the core part in IGFA and the most important improvement of the algorithm in this paper. The task of division is to divide the solution space into G groups. In any group, the dust with the biggest mass value is called the centre dust. The others are called surrounding dusts. A proper division operator will improve the efficiency and reduce the running time.

**Figure 1:**
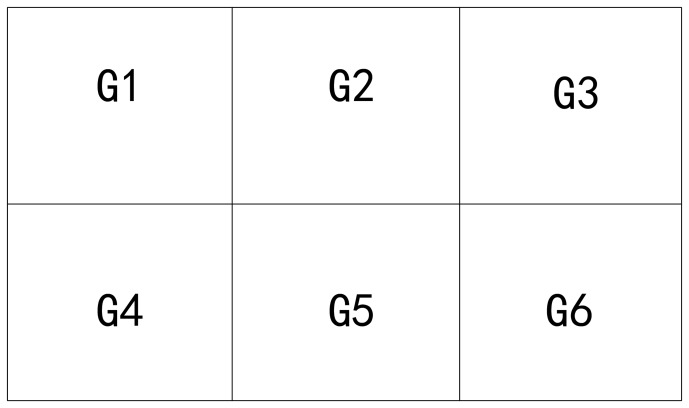
An example of 6 groups with greatest common divisor method. In this method, all areas in solution space are all the same. The number of groups will be decomposed as 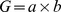. In this example, 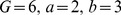.

**Figure 2:**
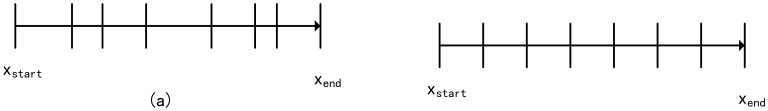
The random method and average method in criteria dimensionality. Every sub-figure in Fig. 2 is divided into 7 groups. Sub-figure (a) is the figure of random division method. In this method, the length of each group is random. Sub-figure (b) is the figure of average division method. In this method, the length of each group is average.

The division strategy in two-dimensional solution space is a smart method which is called the greatest common divisor method described in [6]. An example of 6 groups with this method is shown as Fig. 1. But there is no proper division strategy for higher-dimensional solution space. The easiest strategy is to select one certain dimensionality as division criteria. And other dimensionalities are not considered. The criteria dimensionality, which is a number axis, will be divided with any strategy, such as random method and average method shown as Fig. 2. And other dimensionalities are not the criteria for division.

Although this strategy is feasible for any-dimensional solution space, it is not reasonable for just using one certain dimensionality as division criteria, especially for high-dimensional solution space. So a generic strategy must be proposed in IGFA. Random Division Decomposition (RDD) was proposed in this paper described as following.

**Figure 3:**
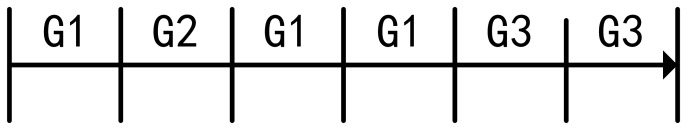
An example of one1dimensional RDD operator. There are 3 groups in this one-dimensional RDD example: G1, G2 and G3. And every dust belongs to these 3 groups randomly. In this example, 1st, 3rd and 4th dusts belong to G1, 2nd dust belongs to G2, and 5th and 6th dusts belong to G3.

**Figure 4:**
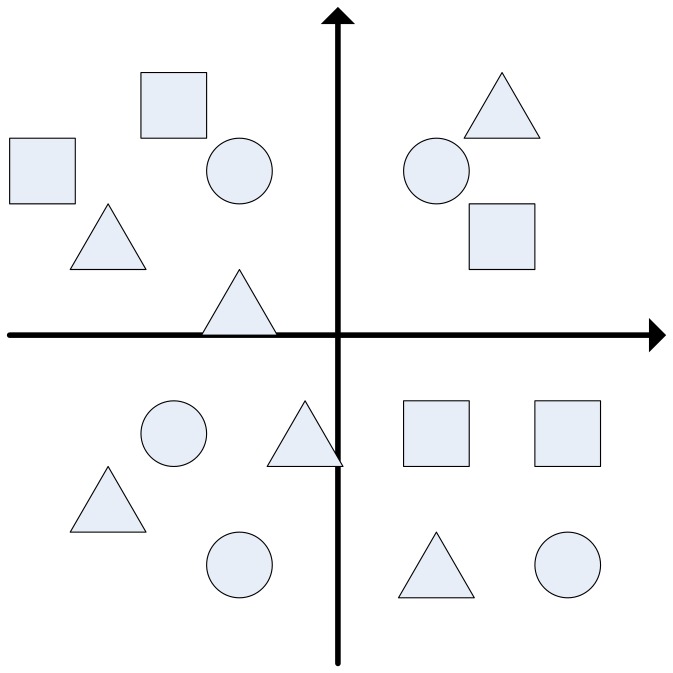
An example of two-dimensional RDD operator. There are 3 groups in this two-dimensional RDD example: G1, G2 and G3. And every dust belongs to these 3 groups randomly. In this example, cycle dusts belong to G1, rectangle dusts belong to G2, and triangle dusts belong to G3.

G is defined as the number of groups in RDD. In one epoch, every dust is allocated to any group i randomly. After membership between all dusts and all groups is determined, movement operator and the corresponding absorption operator will start in this iteration of RDD. RDD operator can be used for any-dimensional solution space, including one-dimensional and higher-dimensional solution space. An example of one-dimensional RDD is shown as Fig. 3. Two-dimensional example is shown as Fig. 4. More information can be seen from Fig. 4. And any dust in RDD just belongs to only one group, not two or more groups. That is, the movement and absorption operators of any dust will only run in their own groups.

Before all movement and absorption operator epochs finish in this iteration, two RDD strategies were designed to change the dusts membership. The first one is called Regular Update Strategy (RUS). In one epoch of RDD, if the optimal value in the whole IGFA procedure is changed in certain number of movement and absorption epochs, the membership of all dusts does not need to update. But if the optimal value in the whole IGFA is not changed at all in certain number of movement and absorption epochs, all groups would be dismissed and every un-deleted dust in solution space will divide randomly again. The main consideration of RUS strategy is that there is no optimal value in all the dusts and the paths along the movement of surrounding dusts. But actually, maybe the optimal value is already in some groups. So another strategy is used in RDD, which is called Never Update Strategy (NUS). In one epoch, no matter the optimal value is changed or not in IGFA, all groups will not be dismissed and updated until this iteration finish.

When the movement and absorption operators in one or more groups finish, the number of dusts in the corresponding groups is just one. And all groups in the whole IGFA will be dismissed. This RDD operation ends. The number of groups G will be updated then. And the membership of all dusts will be determined again. RDD will go into the next epoch.

The selection of RUS and NUS is very important. The algorithm may be not convergent, or the running time will be too long with a wrong selection. A better way is to use a mixed method for RUS and NUS. RUS is used when IGFA is in the earlier period. And NUS is used when GFA is in the later period. It’s hard to decide the border line between these two period, so the number of iterations is used as the criteria to decide. 10 were used for the line between RUS and NUS in the experiments of IGFA.

#### The rules of the movement of the dusts and strategy of absorption

The movement operator is another kernel part in IGFA. The task of this operator is to search the optimal value in each group with absorption operator together. So the convergence of IGFA is related to movement operator, especially when the number of groups is just one in the late period of IGFA. The main idea of this operator is described in the prophase research [6], but some problems should also be resolved. The improved movement operator is described in the following.

The movement is the iterative procedure. In each epoch, the centre dust, whose mass function value is the maximum or minimum in its own group, will be selected at first. The other dusts, which are called surrounding dusts, will be in the gravitation field of the centre dust and move towards centre dust. The rule of movement of surrounding dusts for any-dimensional solution space is mentioned in [6]. But Euclidean distance [12] is easy to calculate only for one-dimensional solution space, which is the difference of two scale number. For multi-dimensional solution space, this method is not an effective one. And much time will be wasted for getting the Euclidean distance value.

In this paper, the distance between two dusts was defined as a difference between two vector variables, which can reduce the running-time of IGFA. For example, the distance between the centre dust (1, 1, 1) and one surrounding dust (3, −1, 7) is (−2, 2, −6), which is also a vector. And this method is called Direct Minus Method (DMM). When the proportion of distance is used for the pace for the movement rules in [6], DMM is the same as the direct Euclidean distance. The reason is described as the mathematical framework section.

**Figure 5:**
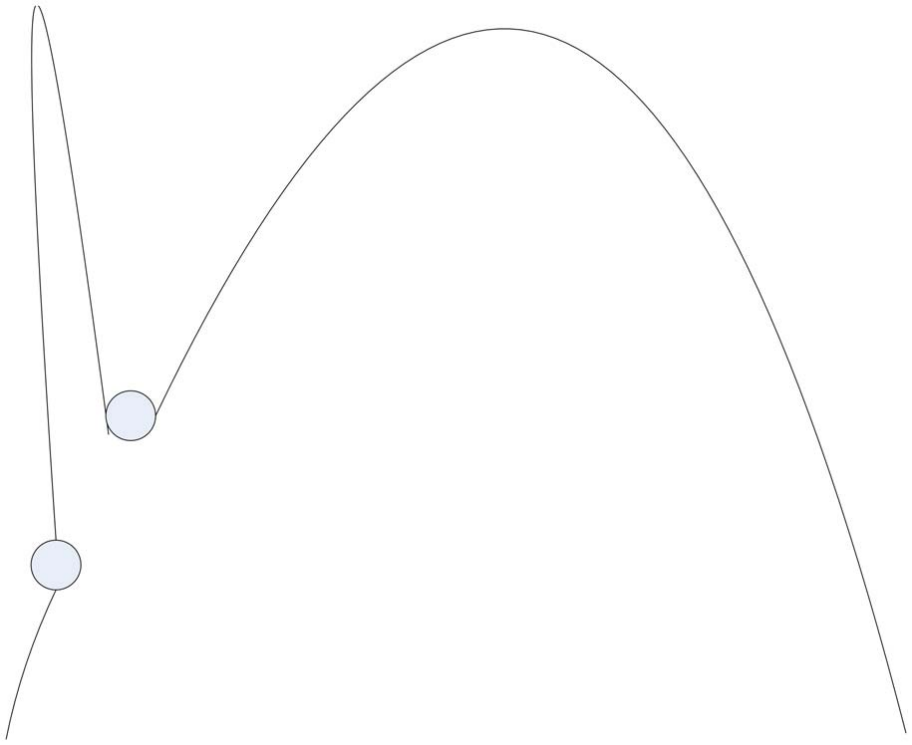
One situation of un-convergence in one-dimensional solution space. The underside cycle was a surrounding dust which didn’t run the movement operator. The upside cycle was the corresponding surrounding dust which had run the movement operator. It was an un-proper movement operator obviously for a bigger pace The optimal value was missed and GFA would not be convergent.

Although the convergence of GFA is proved in three conditions [6], the conditions of infinite dusts can’t be implemented in the computer. One situation of un-convergence in one-dimensional solution space is shown as Fig. 5. So a proper method should be used to increase the convergence probability. The method proposed in this paper is called Rotation Factor (RF) method.

Like the rotation of planets will throw out some dusts, the RF proposed in this paper can be used to prevent local convergence. The rotation operator is used when the movement epoch is completed. The task of rotation is to push surrounding dusts backwards the centre dust. The backward direction is not the original forward direction, but any possible directions randomly. And to avoid too much pace, the max backward pace is defined as Eq. (1):
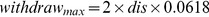
(1)


In Eq. (1), the max backward pace 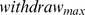 equals 2 times of forward pace which is mentioned in [6]. And dis is not the total length between the centre dust and the original surrounding dust any more, but the current distance.

RF is a probability value with which the rotation operator runs. And RF is inverse proportion to the current distance between the surrounding dust and the centre dust. So the value will change in the whole IGFA process. But only one RF for all dusts will be not proper in IGFA obviously. It’s a better way to set a special RF for each dust. And the RF value is defined as Eq. (2):
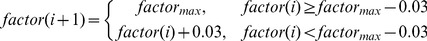
(2)


In Eq. (2), factor (i+1) is the RF after 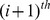 movement operator. 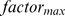 is the max RF. This value can’t be too big, or it will increase running time of IGFA. In this paper, 0.2 was used for this value, and factor(0) = 0. And several times movement operators later, the RF will be the max value.

The basic rules of movement operator are described in detail as above, but a serious problem will be also appeared in movement operator. When a surrounding dust moves towards the centre dust, the location value of the surrounding dust will change to a new one. Obviously, this new value will also be in the solution space in most cases, but it will also be out of the solution space in some cases. So boundary verification should be used to ensure the new value is legal. If the new value is in the solution space, the algorithm will go on, or a new random dust will replace the illegal dust.

The strategy of absorption is easy but efficient. The surrounding dusts will be deleted when the distance between this dust and the centre dust is small enough. And when the number of the surrounding dusts is smaller than the threshold in IGFA, all dusts will be deleted except the centre dust in the group. Then all groups will dismiss and a new iteration of division will begin until the algorithm ends.

The complete pseudo-code of IGFA is presented in Algorithm 1. In Algorithm 1,N is the number of dusts, G is the number of groups. Both of values would decreases continuously in IGFA. So the running-time will be small by this mechanism. ‘GetCentre’ is a method of getting the centre dust through the current dust. And ‘GetMax’ is a method of getting the dust which has the biggest mass value. ‘threshold’ is the smallest distance between the centre dust and the surrounding dust. If the distance is smaller than this value, the corresponding dust will be deleted. centre[] is the final results. It can be one value, or many optima.

**Algorithm 1** GFA

1: 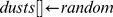


2: **for all** 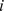 such that 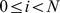 **do**

3: dusts[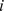] 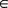 group[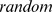]

4: **end for**

5: **for all** 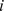 such that 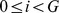 **do**

6: centre[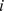] 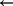 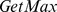
(group[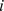])

7: **end for**

8: **end for** 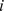 such that 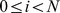 **do**

9: dusts[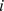] 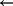 dusts[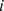]+(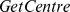
(dusts[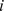])-dust[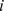])
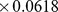


10: **while** dusts[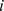]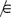 solution space **do**

11: dusts[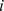]
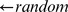


12: **end while**

13: **end for**

14: **for all** 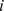 such that 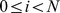 **do**

15: **if** Fun((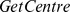
(dusts[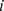]))-Fun(dusts[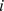]))
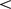
threshold **then**

16: delete dusts[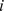]

17: **end if**

18: **end for**

19: **for all** 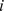 such that 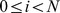 **do**

20: **if** dust[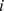] is not 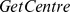
(dusts[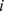]) and 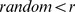 **then**

21: dusts[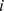] 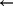 dusts[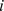]+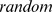


22: **end if**

23: **end for**

24: **if** GroupNotFinish **then**

25: goto [5]

26: **else**

27: goto [29]

28: **end if**

29: **if** Finish **then**

30: return centre[]

31: **else**

32: update N,G

33: goto [2]

34: **end if**

### Mathematical Framework

In this part, DMM can be verified. And a new theorem will be proposed and proved.

#### Theorem 1. the proportions of movement are equal

When the movement operator is on, if the pace is the proportion of the total distance between this dust and the centre dust, the distance of DMM is equal to the Euclidean distance.

If the Theorem 1 for M-dimensional solution space is desired to be proved, two parts must be proved. The first one is that the Theorem 1 is correct when M = 1. The other is that if the Theorem 1 is correct when the solution space is M-dimensional, then Theorem 1 is correct too when the solution space is (M+1)-dimensional. When these two parts are proved, Theorem 1 of any dimensionality can be proved, such as M = 1, 2, 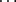. The Theorem 1 will be proved in the theory of Euclidean geometry [13]. We described the proof as following.

#### proof

**Figure 6:**
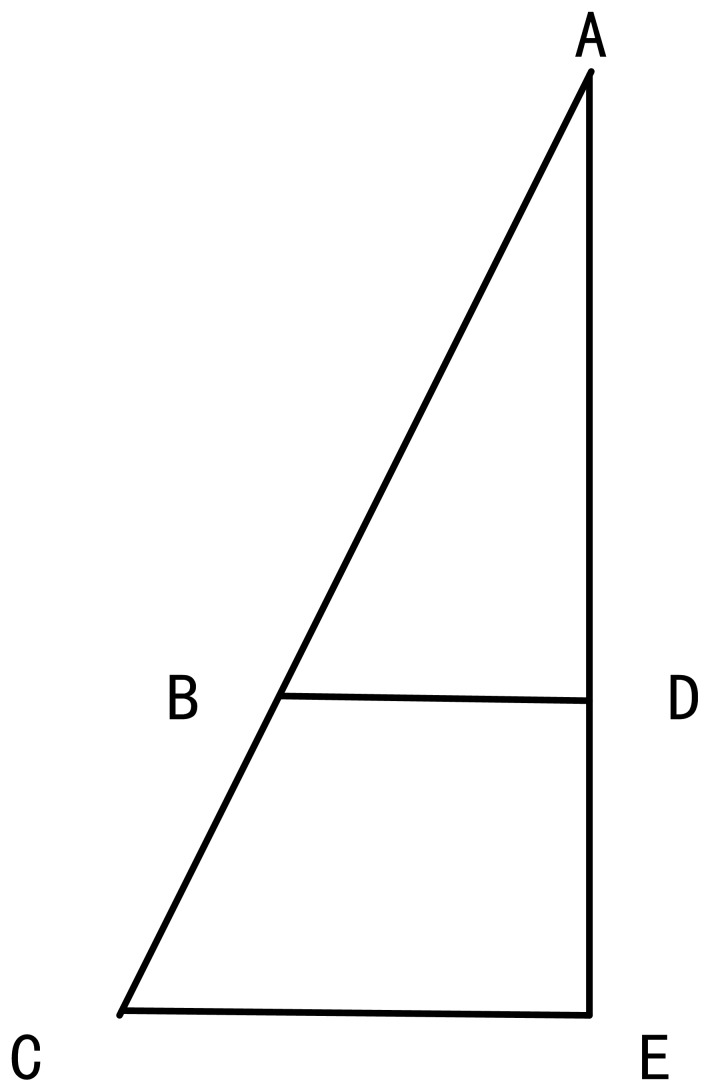
The paragraph of the proportions of movement is equal. AC is the distance of points A and C in (M+1)- dimensional solution space. AE is the projection of AC in M-dimension solution space. CE is the projection of AC in 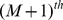-dimensional solution space. AB is one pace between points A and C.

1. The Theorem 1 is correct when M = 1 obviously, since the distance of DMM is the Euclidean distance itself.
2. Fig. 6 is the paragraph of DMM in (M+1) dimension. In Fig. 6, C is the center dust, A is one surrounding dust. The direction of movement is from A to C. The section of line AC is the Euclidean distance of point A and point C in (M+1)-dimensional solution space. The section of line AE is the projection of AC in the M-dimensional solution space. The section of line CE is the projection of AC in the 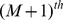-dimensional solution space. The section of line AB is one pace in movement operator between point A and point C. Point D is the projection of point B. So the Theorem 1 is transferred to 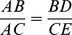 in 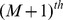-dimensional solution space. Because CE is vertical to the surface of M-dimensional solution space, we can get that 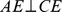. Then 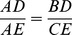 is established. Because Theorem 1 is correct when the number of dimension is M, we can get 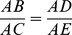. So that 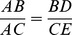. We can get that Theorem 1 is also correct in 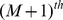-dimensional solution space. Absolutely, we can get that if the Theorem 1 is correct when the solution space is M-dimensional, then Theorem 1 is correct too when the solution space is (M+1)-dimensional. That is the Theorem 1 is correct for any dimensionality.

### The Application to the Hierarchical Clustering

In the theory framework, the mass function within a certain continuous data solution space must be used in IGFA. But for some applications in system biology, especially in hierarchical clustering, the discrete data is used. Thus, the IGFA must be modified again for discrete data in this paper.

First of all, the initial part must be modified. Because all solutions are discrete, a new method should be used for initializing all dusts. The distance function of each genes pair is used as the mass function in IGFA. A series numbers are used for identifying all the dusts, and two numbers are used as parameters in the distance function. In the hierarchical clustering, two integers are selected randomly from 1 to 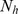
(number of genes) for initialization. One is i, the other is j(j
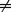
i). The Euclidean distance is used as mass function defined as Eq. (3). i and j are used as dimensionality D in IGFA. And states of all i-j pairs are set to ‘not used’. The number of conditions M is not the dimensionality because it is not the key factor in Eq. (3).
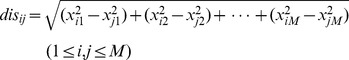
(3)


The other modified part is the movement operator in IGFA. The rule of decrease or increase progressively was used because both i and j were integers in [1, N]. The modified movement operator is described in detail as following:

1. step = 1.
2. If the serial number of centre dust 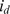 is smaller than the serial number of surrounding dust 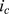, i = i-step. If 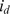 is greater than 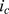, i = i+step. If 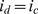, 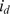 will be not changed. The algorithm goes to (4).
3. If the serial number of centre dust 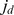 is smaller than the serial number of surrounding dust 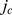, j = j-step. If 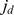 is greater than 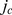, j = j+step. If 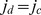, 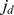 will be not changed. The algorithm goes to (4).
4. If i-j pair of the surrounding dust is the state ‘used’, then step = step+1 and goto (2) or (3) again. If i- j pair is ‘not used’, Then step = 1 and goto (5).
5. After (4), i- j pair must be identified as ‘used’.

The state ‘used’ in (4) and (5) should be used because unexpected results will not appear in the discrete IGFA described above, so the state ‘used’ can reduce the running time of IGFA in the application of hierarchical clustering.

Except these two parts, other parts will not be necessary to modified for the application.

## Results and Discussion

### Test Method

To test the efficiency of IGFA proposed in this paper, a suite of five functions, which include Eq. (4)–(8), was used to assess the algorithm performance. And the test results of IGFA will be summarized and be compared with GFA, GA and SA. 500 different runs of each method and each benchmark function were performed and compared with each other.

Sphere function [14]:
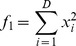
(4)


Rosenbrock function [15]:
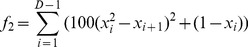
(5)


Rastrigin function [16]:
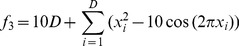
(6)


Griewangk function [17]:
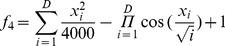
(7)


Ackley function [18]:
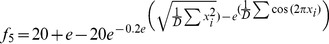
(8)


In Eq. (4)–(8), D is dimensionality. And D = 50 was defined in these experiments. Eq. (4) is the single minimum function, the others can be used as both single minimum and multi minima function.

**Table 1:** Common parameters settings of IGFA, GFA, GA and SA.

Algorithm parameters	IGFA	GFA	GA	SA
Max. numbers of iterations	1000	1000	1000	1000
Population size	50	50	50	–
Number of polulations	200	200	200	–
Initial temperature	–	–	–	0–5.0
various rate	0.3	–	0.3	–

The error functions used to determine the algorithm efficiency were Mean squared error (MSE) [19], Standard deviation (STD) [20] and Mean guass error (MGE) [21] which were defined as Eq. (9)–(11).
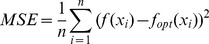
(9)

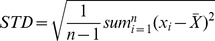
(10)

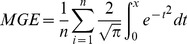
(11)


In these three functions, n is the number of runs, 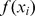 is the performance for run i and 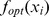 is the real function value at the global minimum.

**Table 2:** MSE, STD, and MGE of IGFA, GFA, GA and SA.

	Sphere	Rosenbrock	Rastrigin	Griewank	Ackley
**IGFA**					
MSE	**0.2854**	0.0143	**78.5732**	**4.6786e-007**	**12.4189**
STD	**0.2847**	**0.0105**	**5.6497**	**4.8973e-004**	12.4189
MGE	**0.4576**	0.0454	**0.7583**	**4.5937e-004**	**1**
**GFA**					
MSE	0.3254	0.0155	151.5743	5.3587e-007	16.4500
STD	0.2931	0.0155	6.5848	5.1176e-004	16.4500
MGE	0.4769	0.1179	0.9587	5.9689e-004	1
**GA**					
MSE	7.2747	**0.0054**	7054.2	6.7709e-007	14.6001
STD	0.7409	0.0556	16.2621	6.8194e-004	**0.1891**
MGE	0.9927	0.0546	1	5.2149e-004	1
**SA**					
MSE	1619.2	0.0069	745.7810	0.0030	26.9123
STD	7.1102	0.0827	12.5530	0.0385	26.9123
MGE	0.9967	**0.0061**	0.9952	0.0439	1

Best performance (i.e., lowest error) for each function is highlighted in bold underline letters.

### Global Minimum Experiment

The common settings of parameters were shown in Table 1. In this table, maximum iteration number is 1000 for all four algorithms. Initial temperature was used just for SA. Various rate for IGFA was the rotation factor, for GA was the mutation rate.

To compare the performance of these four algorithms, 500 different runs of these five functions were used. And the value range of each dimensionality was [−2,2]. The MSE, STD and MGE results had been concluded in Table 2. The efficiency of IGFA could be seen from Table 2.

**Table 3:** Mean numbers of epochs until the minimization threshold was reached and mean number of failures of four algorithms.

	Sphere	Rosenbrock	Rastrigin	Griewank	Ackley
**IGFA**					
mean numberof **epochs**	33	51	36	57	113
number of failures	0	0	0	0	0
**GFA**					
mean number ofepochs	**30**	**47**	**31**	**47**	98
number of failures	0	0	0	0	0
**GA**					
mean numberof epochs	51	57	51	51	**92**
number of failures	0	0	0	0	0
**SA**					
mean number ofepochs	816	46024	6349	21634	1001
number of failures	108	500	314	126	234

**Table 4:** Total running-time of IGFA, GFA, GA and SA with 500 runs.

	Sphere	Rosenbrock	Rastrigin	Griewank	Ackley
**IGFA**	191.2	**62.29**	**103.1**	68.75	**79.57**
**GFA**	201.4	66.84	114.87	67.93	82.58
**GA**	**187.13**	63.29	157.33	**63.98**	101.30
**SA**	14211.08	11463.29	52914.75	15536.02	6406.68

From the comparison results between IGFA, GFA, GA and SA, we could see that for some simple functions, like Sphere’s, Rastrigin’s and Griewank’s functions, MSE, STD and MGE of IGFA were lower than ones of GA and SA. But for some complex functions, like Rosenbrock’s and Ackley’s functions, IGFA was not better than GA and SA.

**Table 5:** MSE, STD, and MGE of IGFA, GFA in searching multi-minima.

	Rosenbrock	Rastrigin	Griewank	Ackley
**IGFA**				
1	**1.0021**	**1.0457**	**0.0101**	3.9940
2	0.1007	**1.0582**	**0.0078**	**3.5018**
3	**0.0021**	**0.0583**	**0.0005**	**0.0024**
4	**0.1037**	**1.0278**	**0.0073**	**3.5370**
5	**1.0026**	0.9587	**0.0075**	3.6948
**GFA**				
1	1.0026	1.0469	0.0117	**3.9842**
2	**0.0997**	1.0943	0.0094	3.5237
3	0.0029	0.0685	0.0009	0.0030
4	0.1043	1.0327	0.0081	3.5797
5	1.0031	**1.0187**	0.0097	**3.6874**

Best performance (i.e., lowest error) for each function is highlighted in bold underline letters.

From Table 2, the efficiency of IGFA could be seen. IGFA was better than GFA in all conditions and for all functions. The STD of IGFA was lower than others for Rosenbrock’s function. Even for the most complex Ackley’s function, MSE of IGFA was lower than all other three algorithms.

**Figure 7:**
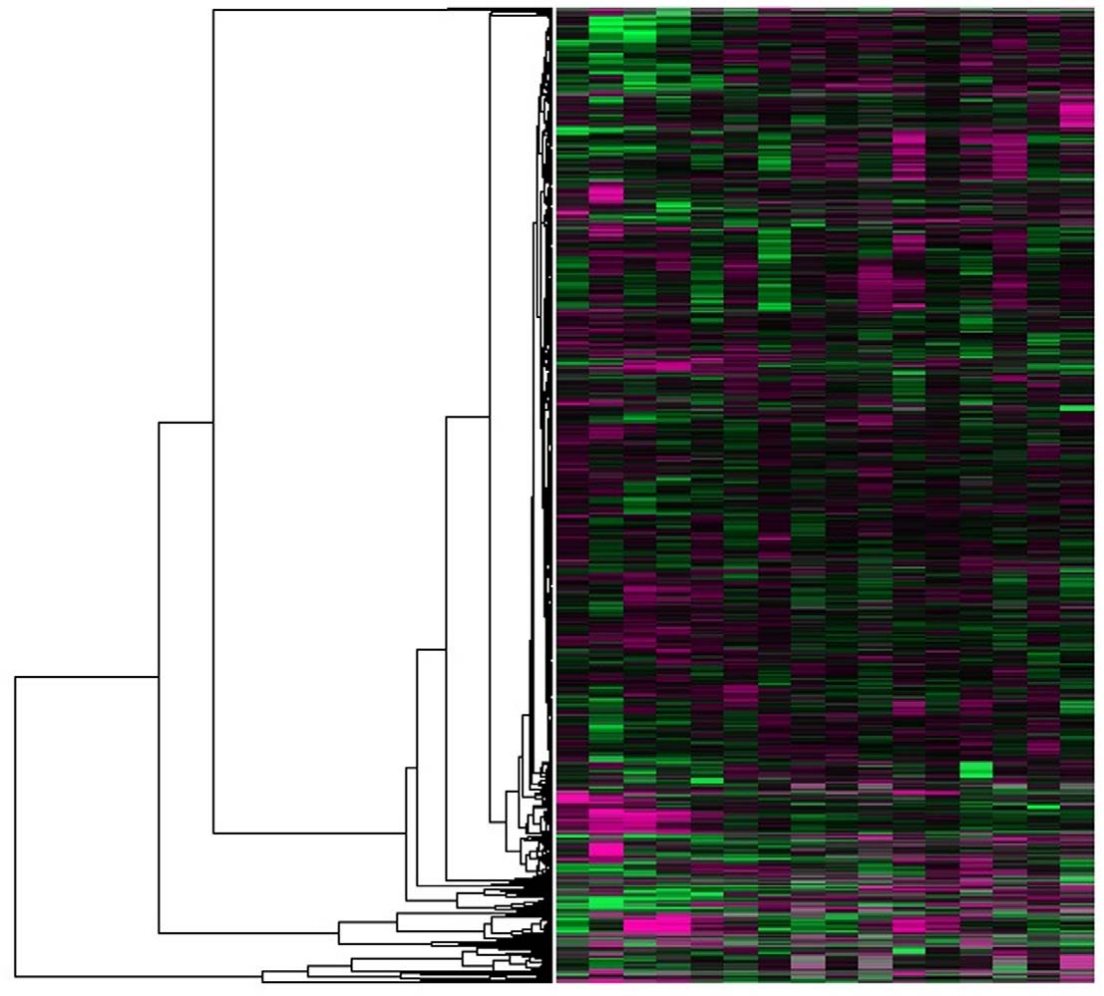
The direct results of hierarchical cluster in GDS38 with IGFA. All the yeast genes were in the hierarchical binary tree. All the relationships between these genes can be seen. The green blocks indicate the low expression. The red blocks indicate the high expression. There are 16 blocks in every row which indicate 16 samples. And there are 7663 rows which state 7663 genes. In addition, the grey blocks indicate the deficit data.

Sphere’s, Rastrigin’s and Griewank’s functions could be optimized better by IGFA. Both Rosenbrock’s and Ackley’s functions could be optimized partly better by IGFA than by others. The lowest criteria were STD and MGE for IGFA. And all functions could be optimized better by IGFA than by GFA.

For running time of IGFA, the rotation factor makes the epochs number bigger, but the rules of random division makes the running time of one epoch smaller. Less time would be used by IGFA to divide the dusts in the solution space.

Time complexity of the four algorithms should be seen both from epoch number and the whole running time of the algorithms. The results had been concluded in Table 3 and Table 4. From Table 3, we could see that the mean epoch number in IGFA with every function was bigger than GFA caused by rotation factor. If IGFA was not convergent within 1,000 epochs, the run was judged as failure. No matter it was convergent or not, the mean epoch number was rounded off and recorded in Table 3. Only SA can reach failure condition.

It seems that only IGFA outperforms for four of five benchmark function. Only for one function, which is Ackley’s function, GA is better than IGFA. But actually, Time complexity of the algorithm was also determined by the running time of one epoch. From Table 4, the total running time can be seen. Rosenbrock’s, Rastrigin’s and Ackley’s functions could be optimized faster by IGFA than other three algorithms. And for Sphere’s and Ackley’s functions, GA will optimized faster.

From the comparison result between IGFA and GFA for all functions, the mean epoch number of IGFA will be bigger than GFA. But total running time of IGFA is smaller than GFA. That is, the efficiency of IGFA is better than GFA.

### Multi-minima Experiment

Although most tasks of given problems is to search the global minimum, multi-minima are also needed by some problems, such as Bayesian network inferring [22]. In this problem, every results is a stochastic network, so a lot of minima will be useful for researchers.

Because the multi-minima searching is beyond GA’s and SA’s ability, the algorithm results of IGFA will be compared with the results with GFA using four benchmark functions: Eq. (5)–(8). Sphere’s function is not suitable for multi-minima searching. In this experiment, the domain in every dimensionality for all four functions was [−2, 2]. The max iteration number was 1000. The number of initial dusts was 30000 for all functions. The number of initial groups was 200. 500 different runs were performed, and 5 minima of two algorithms were calculated and concluded in Table 5.

The error functions were not used. But the direct result values of the multi-valleys searching with IGFA and GFA were calculated and shown in Table 5. And the values in Table 5 were the mean values of 500 different results. In this table, the smallest difference between the calculated value and the real value was in bold.

From Table 5, we could see that for most cases, IGFA could do better than GFA for multi-minima searching functions, including Rosenbrock’s, Rastrigin’s and Griewank’s. And for Ackley function, the efficiency of IGFA was equal to the GFA.

**Table 6:** The compared results table of hierarchical cluster with IGFA, GA, SA, SLC and UPGMA.

algorithms	Distance	running time
**IGFA**	27.34	15.78
**GA**	21.32	19.84
**SA**	11.18	38.74
**SLC**	23.93	78.56
**UPGMA**	21.18	95.78

Distance indicates the mean distance between the minimum child tree which conclude all 300 mitosis genes and the root node of 500 runs. Runing time is the average running time of 500 runs.

### Experiment in Hierarchical Clustering

The data used in this paper for hierarchical clustering is GDS38 in GEO [23] online database. GDS38 is the Yeast Saccharomyces cerevisiae expression data. And the results of the hierarchical clustering will be compared with both GA and SA optimal algorithm, and other traditional hierarchical clustering methods, such as single linkage clustering method (SLC) [24] and unweighted pair-group method with arithmetic means (UPGMA) [25]. And the Euclidean distance is used as criteria function in these algorithms. The data has 7,680 genes with 16 samples, of which the 17 missing values were excluded. So the data used in this paper has 7,663 genes. That is 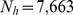 in this experiment.

In traditional hierarchical clustering, 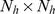 distances must be sorted, it will be wasted to find the minimum, if optimal algorithms can be used in this part, the effective of the algorithm will be proved.

After calculated clustering by IGFA, the direct result is shown as Fig.7 with the free software TreeView [26]. In this red-green heatmap, It is apparent that the similar genes are assigned to the same set (subtree) using a measure of similarity. They almost have the similar color in Fig. 7.

It is impossible to objectively evaluate how good a specific gene expression clustering is without referring to what the gene cluster will be used for. However, once an application has been identified, it may be possible to evaluate objectively the quality of the gene cluster for that special application. In our work, biological applications are desirable. To provide more meaningful biological information, all 300 genes involving mitosis of the yeast cell in the Spellman’s experiment [27] should be near as much as possible in hierarchical clustering result. When conducting the comparisons among different methods for the clustering, it would be good to give some biological significance. A distance between the minimum child tree, which concludes all 300 mitosis genes, and the root node in the binary tree was used as the criterion. An algorithm is good when a distance is long enough. Another parameter for comparing is the running time. To compare the results, 500 runs were performed. And the average results were summarized and shown as Table 6.

The efficiency of the IGFA in hierarchical clustering could be seen from Table 6. IGFA was able to outperform other four algorithms in our experiments, both in accuracy and running time.

In Table 6, the longest distance was the IGFA result. The length was 2.445-fold of SA. That is the most accuracy algorithm was IGFA in the experiments. The accuracy of SLC was in the second place. GA was almost the same as UPGMA. The efficiency of SA was poorly in the experiment. The running-time of any optimal algorithms was lower than the traditional hierarchical clustering methods. And IGFA is the fastest in these three optimal algorithms. The running time of IGFA is lowest, which is 6-fold of UPGMA result.

Overall, The IGFA achieved a longest distance and lowest running time in the experiment. Relatively, the accuracy of any traditional clustering algorithms was in the same magnitude with GA. But the running time is too long to tolerant for clustering.

### Conclusion

In this paper we improved the generic searching-optimization algorithm GFA, which is called IGFA. There are two improved parts in IGFA. One is the rule of random division, which determines the every dust membership. The other is the rule of the rotation factor, which can be used to prevent local convergence. In addition, for the application in hierarchical clustering, IGFA will be modified again to resolve the discrete data problem. The modified parts conclude two parts, one is the initial part, and the other is the movement operator.

Three parts experiments were used in this paper. One is the global minimum searching. In this experiment, the results of IGFA were compared with GFA, GA and SA using five benchmark functions. The second part is the multi-minima searching. The results of IGFA were compared with GFA. The third part is the application in the hierarchical clustering. And the results were compared with GA and SA. The efficiency of the IGFA was proved by these three kinds of experiments.

## References

[1] Simoes R, Emmert-Streib F (2012) Bagging statistical network inference from large-scale gene expression data. Plos One 7. 10.1371/journal.pone.0033624 PMC3316596 22479422

[2] Saunders D, Win J, Cano L, Szabo L, Kamoun S, et al.. (2012) Using hierarchical clustering of secreted protein families to classify and rank candidate effectors of rust fungi. Plos One 7. 10.1371/journal.pone.0029847 PMC3253089 22238666

[3] Zaki N, Bouktif S, Lazarova-Molnar S (2011) A combination of compositional index and genetic algorithm for predicting transmembrane helical segments. Plos One 6. 10.1371/journal.pone.0021821 PMC3144211 21814556

[4] Bank M, Ghomi S, Jolai F, Behnamian J (2012) Application of particle swarm optimization and simulated annealing algorithms in flow shop scheduling problem under linear deterioration. Adv Eng Softw 47: 1–6.

[5] Chuang L, Huang H, Lin M, Yang C (2011) Particle swarm optimization with reinforcement learning for the prediction of cpg islands in the human genome. Plos One 6. 10.1371/journal.pone.0021036 PMC3125183 21738602

[6] Zheng M, Liu G, Zhou C, Liang Y, Wang Y (2010) Gravitation field algorithm and its application in gene cluster. Algorithms for Molecular Biology 5. 10.1186/1748-7188-5-32 PMC2949600 20854683

[7] Wang H, Wang Z, Li X, Gong B, Feng L, et al.. (2011) A robust approach based on weibull distribution for clustering gene expression data. Algorithms for Molecular Biology 6. 10.1186/1748-7188-6-14 PMC3118357 21624141

[8] Zhang X, Moret B (2010) Refining transcriptional regulatory networks using network evolutionary models and gene histories. Algorithms for Molecular Biology 5. 10.1186/1748-7188-5-1 PMC2823753 20047657

[9] Zheng M, Huang Y, Shen W, Zhong Y, Wu J, et al (2012) A novel scale-free network construction method and its application in gene expression profiles simulation. Progress in Biochemistry and Biophysics 39: 581–590.

[10] Safronov VS (1972) Evolution of the Protoplanetary Cloud and Formation of the Earth and the Planets. Moskva: Israel Program for Scientific Translations.

[11] Nebel M, Scheid A, Weinberg F (2011) Random generation of rna secondary structures according to native distributions. Algorithms for Molecular Biology 6. 10.1186/1748-7188-6-24 PMC3354341 21992500

[12] Kurata H, Tarazaga P (2012) Majorization for the eigenvalues of euclidean distance matrices. Linear Algebra Appl 436: 1473–1481.

[13] Peksen O, Khadjiev D, Oren I (2012) Invariant parametrizations and complete systems of global invariants of curves in the pseudo-euclidean geometry. Turk J Math 36: 147–160.

[14] Soler J, Anglada E (2009) Optimal fourier filtering of a function that is strictly confined within a sphere. Comput Phys Commun 180: 1134–1136.

[15] Gurbuzbalaban M, Overton M (2012) On nesterov’s nonsmooth chebyshev-rosenbrock functions. Nonlinear Anal-Theor 75: 1282–1289.

[16] Saez Y, Isasi P, Segovia J (2005) Interactive evolutionary computation algorithms applied to solve rastrigin test functions. Soft Computing as Transdisciplinary Science and Technology 1: 682–691.

[17] Sokolov A, Whitley D, Lunacek M (2005) Alternative implementations of the griewangk function. GECCO 2005: Genetic and Evolutionary Computation Conference Vols 1 and 2: 1589–1590.

[18] Motiian S, Soltanian-Zadeh H (2011) Improved particle swarm optimization and applications to hidden markov model and ackley function. In: 2011 Ieee International Conference on Computational Intelligence for Measurement Systems and Applications (Cimsa). 146–149.

[19] Marchetti S, Tzavidis N (2012) PratesiM (2012) Non-parametric bootstrap mean squared error estimation for m-quantile estimators of small area averages, quantiles and poverty indicators. Comput Stat Data An 56: 2889–2902.

[20] Schoonhoven M, Does R (2012) A robust standard deviation control chart. Technometrics 54: 73–82.

[21] Wan X, Karniadakis G (2006) A sharp error estimate for the fast gauss transform. J Comput Phys 219: 7–12.

[22] Vignes M, Vandel J, Allouche D, Ramadan-Alban N, Cierco-Ayrolles C, et al.. (2011) Gene regulatory network reconstruction using bayesian networks, the dantzig selector, the lasso and their meta-analysis. Plos One 6. 10.1371/journal.pone.0029165 PMC3246469 22216195

[23] Wu J, Liu M, Tsai M, Yu W, Chen J, et al.. (2012) Multi-layer thermoelectric-temperature-mapping microbial incubator designed for geo-biochemistry applications. Review of Scientific Instruments 83. 10.1063/1.4705748 22559585

[24] Sakata S, Ashida F, Tanaka H (2011) Kriging-based convex subspace single linkage method with path-based clustering technique for approximation-based global optimization. Struct Multidiscip O 44.

[25] Gronau I, Moran S (2007) Optimal implementations of upgma and other common clustering algorithms. Inform Process Lett 104: 205–210.

[26] Saldanha A (2004) Java treeview-extensible visualization of microarray data. Bioinformatics 20: 3246–3248. 15180930 10.1093/bioinformatics/bth349

[27] Spellman P, Sherlock G, Zhang M, Iyer V, Anders K, et al (1998) Comprehensive identification of cell cycle-regulated genes of the yeast saccharomyces cerevisiae by microarray hybridization. Molecular Biology of the Cell 9: 3273–3297. 9843569 10.1091/mbc.9.12.3273 PMC25624

